# Bridging the gap: Enhancing pharmacist-physiscian collaboration through the provision of comprehensive medication reviews in community pharmacy

**DOI:** 10.1016/j.rcsop.2024.100555

**Published:** 2024-12-20

**Authors:** Ana I. Sanchez-Molina, Shalom I. Benrimoj, Ramon Ferri-Garcia, Fernando Martinez-Martinez, Miguel Angel Gastelurrutia, Noelia Amador-Fernandez, Victoria Garcia-Cardenas

**Affiliations:** a Pharmaceutical Care Research Group, University of Granada, Spain; b Department of Statistics and Operations Research, University of Granada, Spain; c Graduate School of Health, University of Technology Sydney, Australia; d Pharmacy and Pharmaceutical Technology Department, Clinical, Social and Legal Pharmacy Section, Facultad de Farmacia, Campus Universitario de Cartuja s/n. 18071, Granada, Spain

**Keywords:** Primary health care, Pharmacists, Physicians, Cooperative behaviour, Medication review

## Abstract

**Background:**

Collaborative practice between physicians and pharmacists is particularly important in the provision of pharmacy services, such as Comprehensive Medication Reviews (CMR), which often require a close coordination between these professionals. Understanding the level and nature of this collaboration can assist in the development of strategies to enhance integrated care.

**Objectives:**

(1) To evaluate the changes in level of collaborative practice between community pharmacists and physicians in the context of a CMR service compared to usual practice, from the perspective of community pharmacists, and (2) to explore the determinants of such collaborative practice.

**Design:**

This research was conducted alongside a 12-months prospective longitudinal observational study.

**Methods:**

Community pharmacists providing CMR and pharmacists providing usual care (nCMR) from six provinces in Spain participated in the study. To measure the level of collaborative practice from the perspective of the community pharmacist, a previously validated tool was used. Collaborative practice was assessed at baseline, 6 months and 12 months. A multiple regression analysis was undertaken.

**Results:**

323 pharmacists participated in the study. At the 12-month time point there were statistical differences between CMR and nCMR groups for all factors considered in the level of collaborative practice. Determinants which showed positive significant differences between groups included: rural location of the pharmacy, pharmacists being a pharmacy owner, and female gender of the physician. The age of the pharmacist was negatively associated with higher levels of collaborative practice.

**Conclusion:**

The study provides novel evidence on the level and nature of the collaborative practice between community pharmacists and physicians.

## Highlights

• Collaborative practice between physicians and pharmacists is mainly driven by providing patient-oriented services.
• Comprehensive medication reviews significantly boost collaboration between community pharmacists and physicians.
• Collaboration drivers include pharmacist age, gender, role, physician gender, and pharmacy location.

## 1 Introduction

Collaborative practice between health care professionals was defined by Coluccio M. et al. as: *“Joint communicating and decision-making process with the goal of satisfying the patient's wellness and illness needs while respecting the unique qualities and abilities of each professional”.*^1^ This definition has been widely adopted by many studies studying the collaborative practice between community pharmacists and physicians.^2^^,^^3^ Collaborative practice has been shown to be a cost-effective strategy to improve clinical outcomes^4^^,^^5^ and patients' medication management.^6^^,^^7^

Community pharmacists are increasingly providing patient-centered care, mainly through the provision of professional pharmacy services. Many professional pharmacy services require collaboration between community pharmacists and primary care physicians to provide optimal care,^8^ particularly with chronic diseases management such as asthma,^9^^,^^10^ hypertension,^11^ diabetes^12^^,^^13^ or in medication adherence management.^12^ Different types of medication reviews are a commonly implemented professional pharmacy service, remunerated by Governments in a range of countries such as Australia, Denmark and the United States of America, amongst others.

A consensus document on pharmacy services in Spain identified Comprehensive Medication Review service (CMR) as one of the five professional pharmacy service whose provision and implementation to be prioritised by community pharmacists.^14^ CMR is an ongoing and structured assessment of the patient's pharmacotherapy. It aims at detecting drug-related problems in order to identify, prevent and address negative outcomes related to medicines. This professional service involves a thorough evaluation through a medication review process of a patient's medications to ensure their safety, effectiveness, and appropriateness, focusing not only on ensuring the correct use of medicines but also their expected outcomes in patient's health. In this service, pharmacists are required to work in collaboration with physicians to coordinate a care plan and provide a comprehensive approach to medication management. CMR has been shown to significantly improve clinical outcomes and to be a cost-effective interventions for older adults using polypharmacy.^15^, ^16^, ^17^ During the provision of CMR, pharmacists may interact and collaborate with primary care physicians to jointly address clinical issues relevant to medication management through a shared decision-making process. According to the Collaborative Working Relationship (CWR) framework, one of the most widely used frameworks for guiding collaborative practice between pharmacists and physicians, this collaboration can evolve through a series of stages, potentially reaching a mutually beneficial partnerships with patient-care defined roles and responsibilities. Despite its importance, no literature was found on how the levels of collaboration between community pharmacists and physicians evolve throughout the process of providing professional pharmacy services, such as CMR. This represents as significant gap in understanding the dynamics of collaboration during specific pharmacy services. The provision of CMR require a close cooperation between both professionals to address medication-related problems and negative outcomes associated with medicines, which are key to optimizing patient outcomes. Investigating how collaborative practice develops during in the context of a CMR provision can provide insights into the determinants of successful professional relationships, facilitating the development of targeted interventions to strengthen such practice. Further research is needed to ensure that pharmacy services are delivered in a way that maximizes their clinical impact and facilitate integrated care.The objective of the study was to evaluate the changes in level of collaborative practice between community pharmacists and physicians in the context of a CMR service compared to usual practice from the perspective of community pharmacist. Additionally, determinants of the collaborative practice were examined.

## 2 Method

### 2.1 Study design

This was a prospective longitudinal observational study. To assess the impact of the provision of CMR on the collaborative practice between pharmacists and physicians in the context of the 12-month study, two study groups were considered: those providing CMR (CMR group) and those not providing CMR, which is considered usual practice in Spain (nCMR group).

A longitudinal design was chosen to assess the level of collaboration over time, as CMR implies a close collaboration and continuous patient follow-up. Collaboration between pharmacists and physicians is dynamic, and is expected to evolve as trust, interdependence, perceptions and expectations about the other, skills, interest for collaborative practice, role definition and communication develop in the context of CMR provision. As such, it was hypothesized both professionals would develop an increasing collaborative practice over time.

### 2.2 Collaborative practice assessment

Demographic characteristics were collected at baseline. Aditionally, collaborative practice in both study groups was assessed at baseline, 6 months and 12 months. Each respondent was asked to select a single physician with whom they had professional interactions with and to respond with that physician in mind. To measure the level of collaborative practice from the perspective of the community pharmacist a previously validated tool was used.^18^ This tool is a validated questionnaire which contains 14 items, all answered using a seven-point Likert scale (where 1 refers to “Never”, 2 “Very rarely”, 3 “Rarely”, 4 “Occasionally”, 5” Frequently”, 6 “Very frequently” and 7 “Always”). The 14 items pertain to professional interactions necessary for the development of collaborative practice between physicians and community pharmacists. These items are grouped into three factors^1^: activation for collaborative professional practice (factor 1, consisting of seven items covering physician-pharmacist interactions, max score: 49),^2^ integration of collaborative practice” (factor 2, containing four items reflecting the community pharmacist perception regarding the physician's response to a collaborative approach, max score: 28) and^3^ professional acceptance of collaborative practice (factor 3, containing three items, and reflecting the physician's acceptance of the active role of the community pharmacist in the effectiveness and safety monitoring of medications, max score: 21). An additional single question labelled “collaboration Index”, measured on a scale from zero (minimum level of collaboration) to ten (maximum level of collaboration). This index was used to concisely assess the pharmacist's perceived and self-reported level of collaborative practice. Previous research has shown a strong correlation between the factor scores and this self-reported collaborative index question score.^18^

### 2.3 Study sample

The sample size for the CMR group was determined by the number of pharmacies willing to enroll in a program for the implementation of CMR in community pharmacy in the six participating provinces. The inclusion criteria for study participants were: pharmacy owners and pharmacist employees willing to implement a CMR service. The nCMR group consisted of community pharmacists randomly selected by the local Colleges of Pharmacy from their official list of pharmacies, excluding those pharmacists already allocated to the CMR group. To achieve a 1:1 ratio it was estimated that the number of nCMR pharmacies would be double that of the CMR group due to a potential reduced response rate. The nCMR sample size was 144 pharmacies (with an estimated 216 pharmacists using the Spanish average pharmacists of 1.5 pharmacists per pharmacy). The recruitment process was undertaken by the local Colleges of Pharmacy and researchers did not interfere with it.

### 2.4 Statistical analysis

Participants with a single time point measurement response were eliminated from the analysis. For those who had two of the three measurements, imputation of missing data was undertaken (i.e. replacing that missing value by one that is drawn from an estimate of the distribution of this variable).

Taking only the data of individuals in each group. The CMR group was *n* = 96 and *n* = 82 without imputation and nCMR group was *n* = 99 and *n* = 57 without imputation. The classification and regression tree (CART)^19^ algorithm trained the decision trees on the available data set and imputed the missing value of an individual assigning the value possessed by any of the individuals who shared a terminal node, that is, shared a series of distinctive characteristics. To compare the level of collaborative practice, the difference in means and their standard deviations between study groups for the total summed score of the three factors and each individual factor for each period were used. Student *t*-test for independent samples, applying Holm's correction was used for multiple comparisons in the *p*-values. The analysis compared two scales using the sum of the scores of the Likert scales with and without weighting based on factor loadings and found a correlation of 0.998.

To calculate the scores obtained in the questionnaire and the self-reported score in the collaboration index, Pearson correlations were calculated between the sum of scores in the 14 item and self-reported scores in the collaboration index for each study group and time. To take into account both the variance of the data and the imputations, rules set by Rubin (1991) were applied.^20^

To assess the impact of the individual characteristics on the level of collaborative practice a multivariate analysis was carried out using mixed effects regression models,^21^ with the total sum of the Likert scale scores of the items for each factor as the dependent variable. Three scenarios were considered; scenario 1: Pharmacist as a random effect, scenario 2: Pharmacist and Pharmacy as random effects and scenario 3: Pharmacist, Pharmacy and province as random effects. The residuals fulfilled the hypothesis of normality. The average values of the marginal R2 coefficient and conditional R2 were obtained for the total of 400 combinations and for each of the three models, using the method of Nakagawa and Schielzeth. All analyses were carried out using the statistical software R (R Core Team, 2018).^22^ The libraries tidyverse, mice, Ime4, robustlmm, insight, car and merTools were used.

## 3 Results

**Fig. 1:**
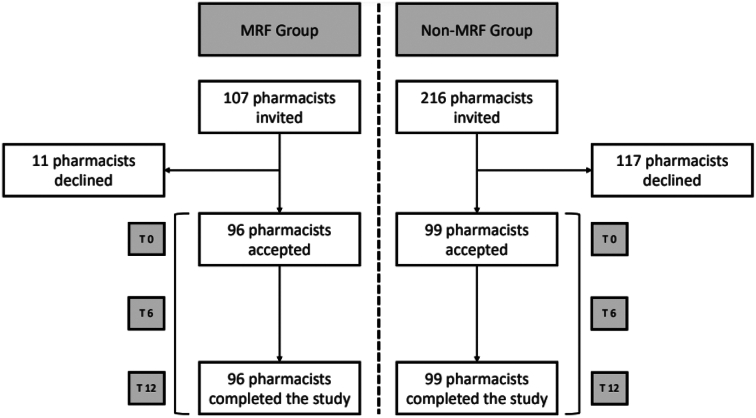
Study Flow Chart.

323 pharmacists were invited to participate in the study (107 in the CMR group and 216 in the nCMR group). The response rate was 89.7 % (*n* = 96) for the CMR group while the response rate for the nCMR group was 45.8 % (*n* = 99) (Table 1 and Fig. 1).

**Table 1:** Characteristics of study sample.

Variable	CMR Group n = 96	nCMR Group n = 99
**Pharmacist age**	41.5 (SD:11.1)	45.9 (SD:11.3)

**Pharmacist gender**		
Female	69 (71.9 %)	68 (68.7 %)
Male	27 (28.1 %)	31 (31.3 %)
**Years of professional practice (pharmacists)**	14.2 (SD:9.7)	18.2 (SD: 10.3)

**Pharmacist role in the pharmacy**		
Pharmacy owner	52 (54.2 %)	69 (69.7 %)
Assistant pharmacist	42 (43.8 %)	28 (28.3 %)
Head pharmacist	1 (1.0 %)	1 (1.0 %)
Other	1 (1.0 %)	1 (1.0 %)

**Working hours**		
Full day (Split schedule)	59 (67.0 %)	66 (74.2 %)
Full day (intensive schedule)	15 (17.0 %)	15 (16.9 %)
Half day, morning shift	4 (4.5 %)	4 (4.5 %)
Half day, afternoon shift	0 (0.0 %)	0 (0.0 %)
Half day, morning shift or afternoon shift	1 (1.1 %)	0 (0.0 %)
Other	9 (10.2 %)	4 (4.5 %)

**Physician gender**		
Female	30 (34.5 %)	33 (36.3 %)
Male	57 (65.5 %)	58 (63.7 %)

**Physician specialty**		
Primary care	87 (98.9 %)	86 (95.6 %)
Practice of physician in public Yes	88 (94.6 %)	85 (88.5 %)
Pharmacy location Rural area	44 (45.8 %)	43 (43.4 %)
Pharmacy location Urban area	52 (54.2 %)	56 (56.6 %)

**Number of employees in the pharmacy**		
Employees 1 to 5	63 (71.6 %)	71 (78.0 %)
Eemployees 6 or more	25 (28.4 %	20 (21.7 %)

**Province**		
Cantabria	13 (13.5 %)	22 (22.2 %)
Toledo	12 (12.5 %)	12 (12.1 %)
Caceres	11 (11.5 %)	10 (10.1 %)
Murcia	22 (22.9 %)	23 (23.2 %)
Zaragoza	14 (14.6 %)	15 (15.2 %)
Leon	24 (25.0 %)	17 (17.2 %)

CMR Group: Community pharmacists group providing Comprehensive Medication Review service.

nCMR Group: Community pharmacist group performing their usual practice in the pharmacy.

The baseline data revealed that the initial level of collaborative practice was low both in the CMR group (average of 33.8 ± 0.61) and in the nCMR group (34.6 ± 0.62)(potential maximum score = 98). There were no statistical differences at baseline between the two study groups for the overall score and for each of the three factors individually (Table 2). At the 6-months time point there was a statistical difference between study groups for factor 1 “Activation for collaborative professional practice” (*p* = 0.008), and for the overall level of collaborative practice (*p* = 0.029). At the 12-months time point there were statistical differences between both study groups for factor 1, “Activation for collaborative professional practice” (*p* < 0.001), factor 2 “Integration of collaborative practice” (*p* = 0.018), factor 3 “Professional acceptance of collaborative practice” (*p* = 0.0185), and for the overall level of collaborative practice (p < 0.001). In the CMR group, there were statistical differences between the scores at the 6-months time point (21.3 ± 0.38) (*p* < 0.002) and at the 12-months time point (23.3 ± 0.43) (*p* < 0.0001) compared to the baseline (15.6 ± 0.41) for all factors. For factor 2 and factor 3 there were also statistical differences between the scores at the 6-month time point and at 12-months compared to the baseline. For the nCMR group no statistical differences were observed when comparing all time points (Table 2). In the self-reported collaboration index (Table 3), no statistical differences between the two study groups were observed at baseline (CMR group (3.32 ± 0.19), nCMR group (3.74 ± 0.21) *p*-value = 0.3665). However, differences were observed at 12-month time points (CMR group (4.91 ± 0.18), nCMR group (3.51 ± 0.21) p-value 0.0075) with statistically higher scores in the CMR group (p-value = 0.0003).

**Table 2:** Mean scores for the three factors and the total sum at baseline (*t* = 0) six-month (*t* = 6) and 12-month (*t* = 12) time points.

Time	Baseline			6 months			12 months		
	CMR Group	nCMR Group	Dif.	CMR Group	nCMR Group	Dif.	CMR Group	nCMR Group	Dif.
**Factor 1**	15.6 ± 0.41	15.1 ± 0.38	0.9564	21.3 ± 0.38	16.6 ± 0.42	**0.0082**	23.3 ± 0.43	15.7 ± 0.54	**<0.001**
Difference to baseline			–	**<0.0002**	1	–	**<0.0001**	1	–
Difference to 6 months				–	–		0.1747	1	
**Factor 2**	8.2 ± 0.23	8.1 ± 0.27	0.8893	10.6 ± 0.28	9.0 ± 0.26	0.1467	11.3 ± 0.31	8.6 ± 0.41	**0.0181**
Difference to baseline			–	**0.0102**	0.4858	–	**0.0008**	1	–
Difference to 6 months				–	–		0.4356	1	
**Fact 3**	10.1 ± 0.23	11 ± 0.26	0.2960	12.2 ± 0.24	11.1 ± 0.26	0.2966	12.4 ± 0.25	10.2 ± 0,31	**0.0185**
Difference to baseline			–	**0.0017**	0.8974	–	**0.0026**	0.0074	–
Difference to 6 months				–	–		0.7560	0.8074	
**Total sum**	33.8 ± 0.61	34.6 ± 0,62	0.7615	44 ± 0.54	36.7 ± 0.62	**0.0298**	46.9 ± 0.66	34.2 ± 0.91	**<0.001**
Difference to baseline			–	**<0.0003**	1	–	**<0.0001**	1	–
Difference to 6 months				–	–		0.2874	1	

CMR Group: Community pharmacists group providing Comprehensive Medication Review service.

nMRF Group: Community pharmacist group providing their usual practice in the pharmacy.

Factor 1: “Activation for collaborative professional practice” (seven items, maximum score = 49).

Factor 2: “Integration of collaborative practice” (four items, maximum score = 28).

Factor 3: “Professional acceptance of collaborative practice” (three items, maximum score = 12).

Total sum: maximum score in the questionnaire (fourteen items, three factors = 98).

**Table 3:** Total sum of level of collaborative practice compared to the self-reported collaborative index.

Time	Baseline			6 months			12 months		
	CMR Group	nCMR Group	Dif.	CMR Group	nCMR Group	Dif. ^1^	CMR Group	nCMR Group	Dif.
**Total sum of level**	33.8 ± 0,61	34.6 ± 0,62	0.762	44.0 ± 0.54	36.7 ± 0.62	**0.0298**	46.9 ± 0.66	34.2 ± 0.91	**0.001**
**Self-reported score**	3.32 ± 0.19	3.74 ± 0.21	0.3665	4.53 ± 0.18	3.73 ± 0.2	**0.1577**	4.91 ± 0.18	3.51 ± 0.21	**0.0075**
Difference to baseline			–	**0.0028**	1	–	**0.0003**	1	–
Difference to 6 months				–	–		0.2880	1	
**Correlation: Total /Self-reported score**	0.799	0.742		0.828	0.832		0.828	0.771	

CMR group: Community pharmacists group providing Comprehensive Medication Review service.

nCMR Group: Community pharmacist group performing their usual practice in the pharmacy.

Total sum of level: maximum score in the questionnaire (fourteen items, three factors = 98).

Self-reported score**:** “Collaboration Index”, measured on a scale from zero (minimum level of collaboration) to ten (maximum level of collaboration).

Determinants of the collaborative professional practice gave similar results in the three scenarios assessed (Tables 4 (scenario 1), 5 (scenario 2) and 6 (scenario 3)). In all the scenarios, participants in the CMR group had higher levels of collaboration by 9.28 (SD:3.96), 9.06 (SD:3.15) and 9.05 (SD:3.15) points at the 6-months time point and 12- months time point by 13.46 (SD:4.65), 13.64 (SD:3.69) and 13.63 (SD:3.69) than the nCMR group. The effect of being in a rural area compared to being in an urban area was estimated to increase the overall score by 9.97 (SD:2.14) (scenario 1), 7.50 (SD:2.59) (scenario 2) and 8.40 (SD:2.41) points (scenario 3). Each year added to the age of the pharmacist represented an estimated average decrease in the level of collaborative practice with varying magnitude in the three different scenarios (Table 4, Table 5, Table 6). In scenario 1 (Table 4), there was evidence that the level of collaboration with the female physicians was higher than with males with an estimated increase of 4.24 (SD:2.02) points. In this same scenario (Table 4), an employee pharmacist compared to a pharmacy owner, had a non-zero effect on the total score with a decrease in score of 5.71 (SD:2.54). In the other scenarios a similar trend was observed with a decrease of 4.35 (SD:2.77) (Table 5) and 4.84 (SD:2.75) (Table 6) points, but these were not significantly different.

**Table 4:** Scenario 1: Robust mixed-effects regression model, pharmacist ID (identifier code of the individual) as random effect.

Variable	Estimate	Std. Error	t-value	p-value
Constant term	31.907	9.638	3.311	**0.0009**
Pharmacist age	−0.162	0.087	−1.874	0.0610
Pharmacist Gender (Female)	0.751	1.966	0.382	0.7025
Full time	−1.360	2.713	−0.501	0.6163
Part time (am)	−3.050	4.562	−0.668	0.5039
Part time (pm)	2.699	16.591	0.163	0.8708
Part time (am or pm)	−4.796	36.716	−0.131	0.8961
Others	0.560	4.818	0.116	0.9075
Employee Pharmacist	−5.709	2.539	−2.248	**0.0246**
Owner pharmacy substitute	17.183	11.222	1.531	0.1258
Other	−7.882	14.350	−0.549	0.5828
Physician Gender = Female	4.237	2.022	2.096	**0.0361**
Physician specialty	6.405	6.727	0.952	0.3412
Physician's professional practice in public and private health care system	6.480	7.450	0.870	0.3844
Physician's professional practice in public health care system	0.758	3.896	0.195	0.8458
Pharmacy area = rural	9.966	2.144	4.648	**<0.0001**
Province = Toledo (45)	5.591	3.602	1.552	0.1206
Province = Cáceres (10)	6.596	3.922	1.682	0.0926
Province = Murcia (30)	4.912	2.829	1.737	0.0825
Province = Zaragoza (50)	6.504	3.173	2.050	**0.0404**
Province = León (24)	7.474	3.170	2.358	**0.0184**
CMR Group	−1.443	3.009	−0.480	0.6315
6 months	1.601	2.728	0.587	0.5573
12 months	−0.750	3.145	−0.238	0.8116
(CMR Group) *(6 months)	9.278	3.985	2.328	**0.0199**
(CMR Group) *(12 months	13.461	4.650	2.895	**0.0038**

CMR Group: Community pharmacists group providing Comprehensive Medication Review service.

Marginal R^2 = 0,2180, conditional R^2 = 0,2984. Goodness-of-fit estimated with the method from Nakagawa and Schielzeth (2013).

Constant term: Mean of the dependent variable when all exploratory variables take value zero.

**Table 5:** Scenario 2: Robust mixed-effects regression model introducing individual ID (identifier code of the individual) and pharmacy ID (identifier code of the pharmacy) as random effects.

Variable	Estimate	Std. Error	t value	p-value
Constant term	33.298	11.327	2.940	**0.0033**
Pharmacist age	−0.144	0.098	−1.477	0.1396
Pharmacist gender = (female)	0.466	2.443	0.191	0.8486
Full time	−1.389	3.677	−0.378	0.7056
Part time (am)	−1.702	5.416	−0.314	0.7534
Part time (pm)	4.987	16.507	0.302	0.7627
Part Time (am or pm)	−2.465	95.855	−0.026	0.9795
Others	2.642	6.292	0.420	0.6745
Employee Pharmacist	−4.347	2.769	−1.570	0.1164
Owner pharmacy substitute	20.924	12.211	1.713	0.0868
Other	−6.459	12.458	−0.518	0.6041
Physician gender = (female)	3.417	2.649	1.290	0.1972
Physician specialist	1.3121	8.151	0.161	0.8721
Physician's professional practice in private health care system	2.596	7.046	0.368	0.7126
Physician's professional practice in public health care system	−2.102	4.048	−0.519	0.6036
Pharmacy area = rural area	7.498	2593	2892	**0,0038**
Province = Toledo (45)	8.694	5.511	1.578	0.1147
Province = Caceres (10)	8.356	5.303	1.576	0.1151
Province = Murcia (30)	7.061	4.430	1.594	0.1110
Province = Zaragoza (50)	7.919	4.917	1.611	0.1073
Province = León (24)	9.120	4.727	1.929	0.0537
CMR Group	−1.806	3.329	−0.542	0.5875
6 months	1.732	2.191	0.791	0.4291
12 months	−0.365	2.552	−0.143	0.8864
(CMR Group) *(6 months)	9.063	3.152	2.876	**0.0040**
(CMR Group) *(12 months)	13.637	3.690	3.696	**0.0002**

CMR Group: Community pharmacists group providing Comprehensive Medication Review service.

Marginal R^2 = 0,1817, conditional R^2 = 0,6026. Goodness-of-fit estimated with the method from Nakagawa and Schielzeth (2013).

Constant term: Mean of the dependent variable when all exploratory variables take value zero.

**Table 6:** Scenario 3: Robust mixed-effects regression model introducing individual ID individual ID (identifier code of the individual) and pharmacy ID (identifier code of the pharmacy), and province as random effects.

Variable	Estimate	Std. Error	t value	p-value
Constant term	40.391	10.888	3.710	**0.0002**
Pharmacist age	−0.164	0.0963	−1.698	0.0895
Pharmacist gender = (female)	0.689	2.437	0.283	0.7773
Full time	−1.490	3.597	−0.415	0.6782
Part time (am)	−1.939	5.325	−0.364	0.7158
Part time (pm)	5.120	16.719	0.306	0.7595
Part time (am or pm) Other	−2.089 2.175	93.342 6.176	−0.022 0.352	0.9822 0.7247
Employee Pharmacist	−4.844	2.749	−1.762	0.0781
Owner pharmacy substitute	20.060	12.272	1.635	0.1023
Other	−7.576	12.433	−0.609	0.5423
Physician gender = (female)	3.272	2.631	1.243	0.2139
Physician specialist	1.318	8227	0.160	0.8727
Physician's professional practice in private health care system	2.688	7.052	0.381	0.7031
Physician's professional practice in public health care system	−1.913	4.070	−0.470	0.6384
Pharmacy area = (rural)	8.386	2.507	3.345	**0.0008**
CMR Group	−1.564	3.322	−0.471	0.6378
6 months	1.750	2.189	0.799	0.4241
12 months	−0.370	3.592	−0.145	0.8850
(CMR Group) *(6 months)	9.052	3.152	2.872	**0.0041**
(CMR Group) *(12 months)	13.634	3.694	3.691	**0.0002**

CMR Group: Community pharmacists group providing Comprehensive Medication Review service.

Marginal R^2 = 0,1540, conditional R^2 = 0,5890. Goodness-of-fit estimated with the method from Nakagawa and Schielzeth (2013).

Constant term: Mean of the dependent variable when all exploratory variables take value zero.

Some significant non-zero effects were observed for some provinces in comparison to the province of reference. In the scenario where the participant was included as a random effect (Table 4), there was an increase in the score if they responded from Zaragoza 6.50 (SD:3.18) points, or Leon 7.47 (SD:3.17) points in comparison to participants from Cantabria. When including the pharmacy as a random effect (Table 5), there was still statistical evidence of a non-zero effect for the province of Leon estimated mean increase at 9.12 (SD:4.73) points.

## 4 Discussion

### 4.1 Key findings

This study provides evidence of the level of collaborative professional practice between community pharmacists and physicians from the perspective of the pharmacist, and the significant positive increases that occur due to the provision of pharmacists-led medication reviews. The baseline data revealed that the current level of collaborative professional practice in Spain is relatively low. This was also confirmed by the self-reporting index where pharmacists were reporting a low level of collaboration with the physician.

### 4.2 Strengths and weaknesses

Interpretation of these results should be tempered by the limitations of the study. The scores for collaborative practice reported were those perceived by community pharmacists in Spain after selecting a single physician. Developing methods and measuring actual collaborative practice behaviour would be worth considering in the future. Additionally, collaborative practice was not measured from the perspective of the physician. Although the nCMR group was randomly selected, their usual practice was assumed not to include CMR. We consider this to be a reasonable assumption considering the general available information of usual practice in Spanish community pharmacies and that CMR is not currently a remunerated service. Moreover, pharmacists included in the CMR group may have been more motivated and willing to provide services like CMR, which may not reflect the general practice of pharmacies in Spain.Another potential limitation of this study is the imputation of missing data, which, while useful for maintaining the sample size, may introduce bias. Nevertheless, imputation was only undertaken for those participants who had two of the three measurements, by replacing that missing value by one that was drawn from an estimate of the distribution of this variable, which is considered a valid and commonly accepted method.

Finaly, the internal and external validity of this research may be compromised due to the participants selection bias. Pharmacists in the CMR group self-selected for the study and may not be representative of Spanish pharmacists. Nevertheless, the use of a validated tool to measure the level of collaborative practice ensures our results are valid and reliable.

### 4.3 Interpretation

Significant differences in the level of collaborative professional practice were observed in the CMR group, with increased scores for the factor 1 (“Activation of collaborative professional practice”), at 6-months and at 12-months' time points with respect to the baseline. However, a reduced magnitude of the increased level of collaboration was observed from 6 to 12 months, when compared to baseline to 6 months. This suggests that although reaching a level of collaboration during the initial stages of the collaborative practice might be easier, progressing to a deeper level requires more time and probably more in-depth interactions. The individual analysis of the individual factors composing the tool allowed the identification of which interactions are more achievable at the initiation of the collaborative relationship and which ones may present a major challenge. Findings could be used to facilitate the development of strategies and educational programs aimed at improving the collaborative practice.

A systematic review identified a range of collaborative practice models, with the “Collaborative working relationship between community pharmacist and physician” (CWR),^23^ being the most cited. Our findings align with the concepts proposed by Doucette et al. (2001)^23^ where the preliminary interactions of a collaborative practice are initiated by the pharmacist. However, as time progresses these become bilateral and more complex. During the 12-month study, the larger increases in scores were observed for factor 1, which was associated with simple and unilateral interactions. The more complex interactions were measured through factor 2 and 3, and although significant differences were found, the magnitude was lower. However, our study findings do not support a staged approach model,^23^ but rather suggest a continuous improvement of collaborative practice over time. Instead of indicating an evolution through different collaboration stages, our results show that an increase in collaborative practice leads to a higher frequency of interactions between pharmacists and physicians. Increases in factor 2 scores (which measured trust and communication) and factor 3 (which measured role acceptance), were in agreement with the findings from previous reviews.^24^^,^^25^ The provision of a CMR service enhances these elements improving the collaborative practice between both professionals.

The overall questionnaire and each factor score for the CMR group achieved during the 12 months was approximately 50 % of the achievable scores. Equally, changes observed from 6-months to 12-months' time points were lower compared to those observed from the baseline to the 6-months' time point. Prolonging the study might have achieved higher scores. However, to improve the collaborative practice between community pharmacists and physicians, additional effective interventions might be required. A range of strategies have been described in the literature, including co-education of both health care professionals or the application of technologies such as shared IT programs with clinical data and communication programs, that enable an increase the competencies for collaborative practice.

CMR may have had a positive effect on collaboration on the physicians due to an increased communication, and a positive perception of the competencies and capabilities of the pharmacist, thereby instilling an increased confidence and trust. Bollen et al.^25^ carried out a systematic review and classified the factors that influenced collaborative practice into four groups: “Negotiating professionals boundaries”, “Perceived skills and knowledge”, “Structural and organisational facilitators” and “Training and education”, which were consistent with the findings from previous reviews. Within these classifications, the critical elements that moderate the collaborative practice were communication, trust and role acceptance. The most frequent facilitator reported in the literature was increased communication. Additionally, they indicated that an increased awareness of pharmacists´ competencies by physicians was important in engaging in collaborative practice. Bollen et al.,^25^ McDonough et al.,^23^ and Bardet et al.^24^ all agree that the patient-oriented role of the pharmacist, achieved in our study through the provision of CMR, is an essential facilitator of collaborative practice. Nevertheless, the level of collaborative practice can significantly differ between different pharmacy services. This is mostly attributable to their nature and ultimate objective, which influences the type and frequency of interprofessional interactions. The tool used to measure the level of collaborative practice could be validated to measure the impact of additional professional services on the level of collaborative practice, such as minor ailments, adherence management or new medicine services, amongst others.

In this study, three scenarios were proposed to assess how the pharmacist, the pharmacy and the province influenced the level of collaborative practice. Three scenarios were proposed to evaluate the effect of each predictor (i.e. pharmacist, the pharmacy and the province) in the presence of the rest, thus avoiding the phenomenon of confusion that could appear when the association observed between a predictor and the response variable is explained by another variable.

The determinants found to be significantly associated with an increased collaborative practice, included the pharmacist's age, gender, being a pharmacy owner and the rural location of the pharmacy. In their original CWR paper, McDonough et al.^23^ suggested those individual characteristics that affect an individual predisposition to collaborative practice. They hypothesized that younger health care practitioners who may have been more exposed to interdisciplinary education may be more likely to engage in collaborative practice. However, Doucette et al.^26^ found no significant differences in individual characteristics including age, gender, being a pharmacy owner and setting affecting collaborative practice. This may have affected Bardet et al.^24^ theoretical meta-model findings, which highlighted that the influence of individual characteristics was “ambiguous”. Findings from this study suggest that age may negatively affect the extent of collaborative practice. Other influencing factors may be social norms, power perceptions and hierarchy. It was also found that the gender of the physician was perceived to be a positive characteristic for collaborative practice, which has not previously been reported in the literature. Co-location of pharmacists and physicians has been identified in both theoretical models and empirical studies as a facilitator for collaborative practice. In our study, the rural location of the pharmacy was found to be a determinant for increased collaboration, probably due to a closer distance, and regular and more frequent contacts between both health care providers. Finally, when compared to employees, pharmacy owners had a higher level of collaborative practice. Although Doucette et al.^26^ did not find any differences; they did hypothesise that this might have an effect in the level of collaborative practice. In our study the reasons for this to occur are yet to be explored, but it is likely to be affected by Spanish pharmacy ownership rules, where a pharmacist can only own one pharmacy.

### 4.4 Further implications

Our results have demonstrated a strong correlation between the collaboration index and the scores obtained through the professional collaborative practice tool. From a practical perspective, this suggests that the collaboration index could serve as a feasible, fast, and efficient method for self-evaluating collaborative practice. This approach would be useful in busy and pressured primary care settings, where time and resources are limited. However, there may be a need to increase awareness amongst both community pharmacists and physicians regarding the types of interactions that define and drive collaborative practice and how these interactions can be enhanced. Educating healthcare professionals about these key interactions could further strengthen integrated care, ultimately leading to improved patient care and more effective pharmacy services provision.

## 5 Conclusions

This study found that pharmacists providing CMR have higher levels of collaborative practice with physicians. The longitudinal nature of the study allowed the identification of factors strengthening the collaboration and those needing further development. Future studies on collaboration between community pharmacists and physicinas could evaluate the evolution of the three factors driving collaborative practice within the context of other professional pharmacy services. This would be valuable for developing individualized strategies to improve collaborative practice tailored to each professional service. In addition, collaborative practice from the perspective of the physicians could also be investigated using the same approach.

## Funding

This work was supported by the Spanish General Council of Official Colleges of Pharmacists through a grant from Cinfa Pharmaceuticals. Neither of these organisations influenced the study design, interpretation of data, writing of the manuscript, nor the decision to submit this manuscript for publication. The Spanish General Council of Official Colleges of Pharmacy assisted with the selection of the study locations and contacting community pharmacies (contract number 21/1/204 UGR.CGCOF).

## Ethical considerations

Ethical approval (approval number 13/C-11) was provided by the Ethics and Research Committee of the Virgen de las Nieves University Hospital in Granada, with registration number (357/CEIH/2017) Spain. An information sheet was provided, and informed consent was obtained from all participants.

## CRediT authorship contribution statement

**Ana I. Sanchez-Molina:** Writing – original draft, Methodology, Investigation, Conceptualization. **Shalom I. Benrimoj:** Writing – original draft, Conceptualization. **Ramon Ferri-Garcia:** Formal analysis. **Fernando Martinez-Martinez:** Conceptualization. **Miguel Angel Gastelurrutia:** Writing – original draft, Conceptualization. **Noelia Amador-Fernandez:** Conceptualization, Writing – original draft, Writing – review & editing. **Victoria Garcia-Cardenas:** Supervision, Methodology, Conceptualization.

## Declaration of competing interest

The authors have no conflict of interest to declare.
